# Beta-blockade improves right ventricular diastolic function in exercising pulmonary arterial hypertension

**DOI:** 10.1183/13993003.00144-2023

**Published:** 2023-05-18

**Authors:** Chinthaka B. Samaranayake, Aleksander Kempny, Robert Naeije, Michael Gatzoulis, Laura C. Price, Konstantinos Dimopoulos, Lan Zhao, Stephen J. Wort, Colm McCabe

**Affiliations:** 1 National Pulmonary Hypertension Service, Royal Brompton Hospital, London, UK; 2 National Heart and Lung Institute, Imperial College, London, UK; 3 Free University of Brussels, Brussels, Belgium

## Abstract

Right ventricular (RV) function is a main determinant of outcome in pulmonary arterial hypertension (PAH) [1]. Increased neuro-humoral activation associated with decreased survival in PAH may worsen RV adaptation to increased afterload [2, 3]. There is rationale therefore for the use of β-blockers in PAH, especially given their beneficial effect on RV function in experimental PAH models [4, 5]. Small clinical studies in PAH using bisoprolol and carvedilol have demonstrated acceptable patient tolerance, however no change in exercise capacity was seen and, concerningly, resting cardiac output (CO) decreased despite unchanged or even mild improvement in RV ejection fraction (EF) [6, 7].

## Abstract 2

**Beta-blockade improves right ventricular exercise diastolic function in pulmonary arterial hypertension *via* improved heterometric adaptation to the increase in venous return. Further studies are required to determine mechanisms underlying this change.** https://bit.ly/3ZMT8bu

*To the Editor*:

Right ventricular (RV) function is a main determinant of outcome in pulmonary arterial hypertension (PAH) [1]. Increased neuro-humoral activation associated with decreased survival in PAH may worsen RV adaptation to increased afterload [2, 3]. There is rationale therefore for the use of β-blockers in PAH, especially given their beneficial effect on RV function in experimental PAH models [4, 5]. Small clinical studies in PAH using bisoprolol and carvedilol have demonstrated acceptable patient tolerance, however no change in exercise capacity was seen and, concerningly, resting cardiac output (CO) decreased despite unchanged or even mild improvement in RV ejection fraction (EF) [6, 7]. Moreover, in exercising PAH, associated with impaired RV adaptation to high afterload, acute effects of β-blockers on the RV are unstudied. In the present study, we reasoned that the acute administration of β-blockers in PAH patients might improve RV diastolic filling, as has been observed in experimental PAH [5], and accordingly contribute to preservation of coupling of RV function to the pulmonary circulation during exercise.

Eight patients (six men and two women) with idiopathic PAH referred for routine clinical right heart catheterisation at the National Pulmonary Hypertension Service, Royal Brompton Hospital gave informed written consent to the study, which was approved by the local human research ethics committee (17/LO/1686). None of them had a resting heart rate <50 beats per minute or were taking anti-arrhythmic medications. Relevant comorbidities included moderate obstructive sleep apnoea well controlled on positive pressure therapy in two patients. All patients agreed to additional insertion of conductance catheters for instantaneous measurements of RV pressures and volumes, and to the *i.v*. administration of the short-acting β_1_-blocker esmolol during low workload exercise stress.

Right heart catheterisation for measurements of pulmonary artery pressure (PAP), wedged PAP (PAWP), right atrial pressure (RAP) and thermodilution CO was performed using a fluid-filled thermodilution catheter according to current guidelines [8]. Additional catheterisation of the RV was performed using a 7-Fr high-fidelity conductance catheter (CD Leycom, Zoetermeer, the Netherlands), with measurements of instantaneous pressures and volumes as previously reported [9].

The patients were positioned on an electronically braked lower limb cycle ergometer attached to the laboratory table in supine position. Standard pulmonary haemodynamic measurements were obtained. The fluid-filled catheter was withdrawn and replaced by the conductance catheter. After re-calibration of the equipment, RV pressure–volume loops were recorded first at rest, then after three periods of 3 min of exercise at constant 25 W workload successively without drug and with continuous infusions of esmolol at rates of 50 and 100 μg·kg^−1^·min^−1^. Esmolol (AOP Orphan Pharmaceuticals AG; Purkersdorf, Austria) was diluted to a concentration of 2 mg·mL^−1^ in isotonic saline. Each exercise period was resumed after heart rate (HR) had returned to within 10% of baseline values.

Systolic RV function was assessed by the RV stroke work index, maximum rate of isovolumic pressure increase (dP/dt_max_), EF and end-systolic elastance (Ees). Diastolic function was assessed by maximum rate of isovolumic pressure decline (dP/dt_min_) and tau (τ), the time constant of isovolumic relaxation. Tau (τ) represents the time constant of isovolumic pressure decay and is measured during active myocardial relaxation and is calculated as a parameter in an exponential fit to the pressure channel data (Weiss' method), using the following equation: P(t)=A·exp(−t/τ), where t is time and A is the fitted parameter. Single beat estimation of Ees was carried out using a sinusoidal curve fit algorithm written to estimate theoretical maximum isovolumic pressure (P_max_) from dP/dt_max_ and dP/dt_min_ during ventricular ejection [9].

Continuous variables were summarised as mean±sd or median and interquartile range (IQR). Between groups comparison employed independent sample Kruskal–Wallis tests with *post hoc* pairwise comparisons performed using the Mann–Whitney test. Pre- and post-intervention comparisons were performed using paired sample Wilcoxon signed rank test. A p-value of <0.05 was used to establish statistical significance. A power calculation was based on the findings of previous studies describing changes in RV function during exercise in PAH [10, 11] showed that eight patients were required to detect a 10% change in RV end diastolic volume (EDV) (80% power, 5% alpha). Statistical analyses were performed using SPSS Statistics V27 (IBM Corp., Armonk, NY, USA).

The median age of the patients was 51 years (IQR 21 years), and all were in World Health Organization (WHO) function class II (n=5) or III (n=3). Six patients were established on pulmonary vasodilator therapy, either phosphodiesterase-5 inhibitors or endothelin receptor antagonists or both, and three were also treated with intravenous prostacyclin. Median baseline haemodynamics were as follows: HR 67 bpm (IQR 23 bpm), mean arterial blood pressure 88 mmHg (IQR 19 mmHg), RAP 5 mmHg (IQR 5 mmHg), mean PAP 35 mmHg (IQR 22 mmHg), PAWP 6 mmHg (IQR 3 mmHg), CO 4.2 L·min^−1^ (IQR 0.8 L·min^−1^), pulmonary vascular resistance 7.3 Wood units (IQR 5.7 Wood units).

Measurements of RV function at rest and during exercise with or without esmolol are summarised in figure 1. At baseline, stroke volume index (SVi), EF and Ees/Ea were decreased while Ees, arterial elastance (Ea), dP/dt_min_ and τ were increased. End-systolic volume index (ESVi), EDV index (EDVi) and end diastolic pressure (EDP) remained within limits of normal. Exercise was associated with an increase in HR, a decrease in SVi, ESVi and EDVi, unchanged EF, increased Ees and Ea, but decreased Ees/Ea. dP/dt_min_, τ and EDP all increased. Esmolol during exercise decreased HR, restored SVi and brought about a slight decrease in EF despite minimal influence on Ees, Ea and Ees/Ea. Esmolol also restored dP/dt_min_ and τ with a marked increase in EDVi during exercise and return in EDP to baseline. dP/dt_max_ was unaffected by esmolol. The increase in absolute EDVi (figure 1d) during esmolol infusion was dose-dependent and closely associated with HR attenuation (r=−0.81, p=0.012). Stroke work did not change between the exercise phases of the study with median of 1596 mmHg·mL (IQR 542 mmHg·mL) at rest, 2380 mmHg·mL (IQR 1138 mmHg·mL) with exercise, 2207 mmHg·mL (IQR 1097 mmHg·mL) with exercise plus esmolol (0.05 mg·kg^−1^·min^−1^) and 2249 mmHg·mL (IQR 794 mmHg·mL) with exercise plus esmolol (0.1 mg·kg^−1^·min^−1^).

**FIGURE 1:**
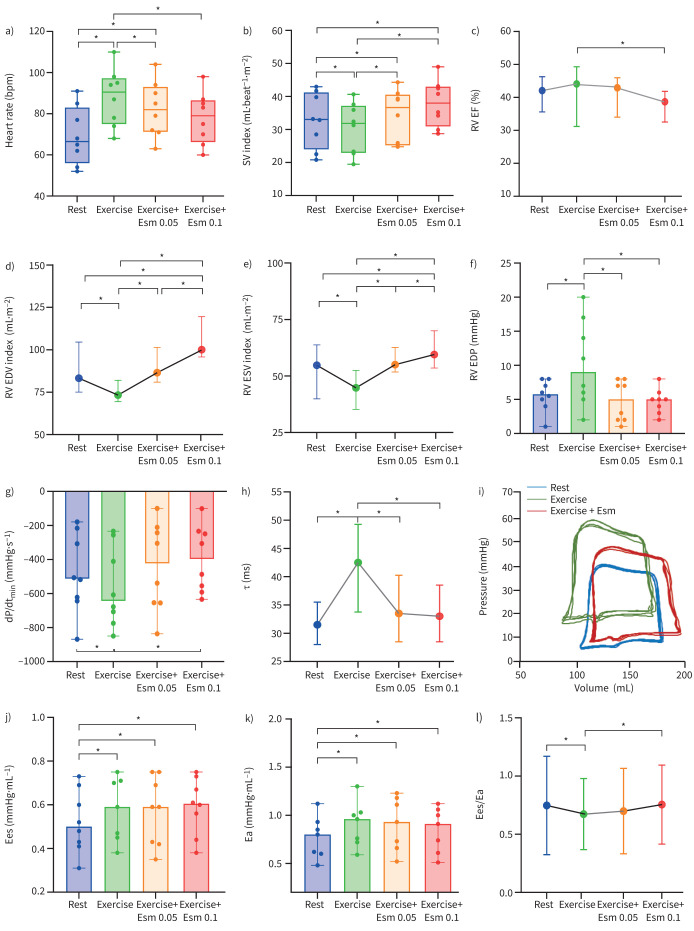
Individual haemodynamic changes in eight patients undergoing pressure–volume catheterisation of the right ventricle (RV) at rest and during exercise with esmolol infusion. Data shown includes transition between rest, exercise, exercise with esmolol (Esm) 0.05 mg·kg^−1^·min^−1^ and exercise with esmolol 0.1 mg·kg^−1^·min^−1^ in a) heart rate (beats per min (bpm)), b) stroke volume (SV) index (mL·beat^−1^·m^−2^), c) RV ejection fraction (EF) (%), d) RV end diastolic volume (EDV) index (mL·m^−2^), e) RV end systolic volume (ESV) index (mL·m^−2^), f) RV end diastolic pressure (EDP) (mmHg), g) maximum rate of isovolumic pressure decline (dP/dt_min_) (mmHg·s^−1^), h) time constant of isovolumic pressure decay (τ) (ms), i) representative pressure–volume loops in a single patient, j) RV elastance (Ees) (mmHg·mL^−1^), k) pulmonary arterial elastance (Ea) (mmHg·mL^−1^), and l) RV arterial coupling (Ees/Ea). *: p<0.05.

The present results confirm previously reported exercise-induced increase in Ea and insufficiently matched increase in Ees resulting in RV–pulmonary artery uncoupling as assessed by decreased Ees/Ea in PAH patients [10, 11]. The selective β1 receptor blocker esmolol did not affect indices of exercise RV systolic function except for a slight decrease in EF. This builds on findings of previous experimental studies showing that the non-selective β-blocker propranolol decreased the Ees/Ea ratio only by a combination of slight and non-significant decrease in Ees and increase in Ea [12]. Mild uncoupling of RV systolic function from the pulmonary circulation by acute or short-term β-blocker administration at rest may therefore not be a matter of clinical concern.

The striking novelty of the present findings was the marked improvement in RV diastolic function by the administration of esmolol during exercise, thus allowing for heterometric adaptation of the afterloaded RV to the exercise-induced increase in venous return. The present data do not allow assessment of the functional consequences of this finding in PAH as no maximum exercise stress test was performed. However, the magnitude of esmolol-related improvement in RV diastolic function deserves further scrutiny, especially since it has been shown that increased diastolic stiffness in PAH may be dissociated from systolic elastance [13], and independently predicts mortality [14].

The present findings are limited by the small size of the study, mildly abnormal pulmonary haemodynamics in a prevalent patient cohort receiving treatment for PAH with preserved WHO functional class and observations of only acute effects of a selective β_1_-blocker. However, exercise stress testing revealed marked diastolic changes, which has also been reported in experimental PAH in resting conditions [5]. Whether this is a simple consequence of a slowing of HR with a secondary influence on increased RV diastolic filling [5], or related to specific metabolic effects of different β-blocker classes [15], remains to be clarified. It is also uncertain how acute changes in RV diastolic filling during exercise may translate to chronic β-blocker administration given potential effects on the RV unstressed volume. Given these limitations, our results should therefore be regarded as hypothesis generating. Thus, confirmation with longer-term administration of different β-blockers is needed to determine β-blocker class-selective effects on clinical improvement.

## Shareable PDF

10.1183/13993003.00144-2023.Shareable1 This one-page PDF can be shared freely online.
Shareable PDF ERJ-00144-2023.Shareable
