# Supplementary material for: Beta-blockade improves right ventricular diastolic function in exercising pulmonary arterial hypertension
Source: Eur Respir J. 2023 May 18;61(5):2300144. doi: 10.1183/13993003.00144-2023 (PMC10196346; doi:10.1183/13993003.00144-2023)
Supplement: Supplementary file 1 [file ERJ-00144-2023.Shareable.pdf]

SHAREABLE PDF

Beta-blockade improves right ventricular diastolic function in exercising pulmonary arterial hypertension

Chinthaka B. Samaranayake[1], Aleksander Kempny[1,2], Robert Naeije[3], Michael Gatzoulis[1,2], Laura C. Price[1,2], Konstantinos Dimopoulos[1,2], Lan Zhao[2], Stephen J. Wort[1,2] and Colm McCabe[1,2]

[1]National Pulmonary Hypertension Service, Royal Brompton Hospital, London, UK. [2]National Heart and Lung Institute, Imperial College, London, UK. [3]Free University of Brussels, Brussels, Belgium.

Corresponding author: Colm McCabe (c.mccabe2@rbht.nhs.uk)

Check for updates

Shareable abstract (@ERSpublications)
Beta-blockade improves right ventricular exercise diastolic function in pulmonary arterial hypertension *via* improved heterometric adaptation to the increase in venous return. Further studies are required to determine mechanisms underlying this change. https://bit.ly/3ZMT8bu

Cite this article as: Samaranayake CB, Kempny A, Naeije R, *et al*. Beta-blockade improves right ventricular diastolic function in exercising pulmonary arterial hypertension. *Eur Respir J* 2023; 61: 2300144 [DOI: 10.1183/13993003.00144-2023].

This single-page version can be shared freely online.

Copyright ©The authors 2023.

This version is distributed under the terms of the Creative Commons Attribution Licence 4.0.

This article has an editorial commentary: https://doi.org/10.1183/13993003.00509-2023

Received: 22 Jan 2023
Accepted: 01 March 2023

To the Editor:

Right ventricular (RV) function is a main determinant of outcome in pulmonary arterial hypertension (PAH) [1]. Increased neuro-humoral activation associated with decreased survival in PAH may worsen RV adaptation to increased afterload [2, 3]. There is rationale therefore for the use of β-blockers in PAH, especially given their beneficial effect on RV function in experimental PAH models [4, 5]. Small clinical studies in PAH using bisoprolol and carvedilol have demonstrated acceptable patient tolerance, however no change in exercise capacity was seen and, concerningly, resting cardiac output (CO) decreased despite unchanged or even mild improvement in RV ejection fraction (EF) [6, 7]. Moreover, in exercising PAH, associated with impaired RV adaptation to high afterload, acute effects of β-blockers on the RV are unstudied. In the present study, we reasoned that the acute administration of β-blockers in PAH patients might improve RV diastolic filling, as has been observed in experimental PAH [5], and accordingly contribute to preservation of coupling of RV function to the pulmonary circulation during exercise.
